# Expression of APOBEC3G/3F and G-to-A Hypermutation Levels in HIV-1-Infected Children with Different Profiles of Disease Progression

**DOI:** 10.1371/journal.pone.0024118

**Published:** 2011-08-29

**Authors:** Nívea D. Amoêdo, Adriana O. Afonso, Sílvia M. Cunha, Ricardo H. Oliveira, Elizabeth S. Machado, Marcelo A. Soares

**Affiliations:** 1 Instituto de Bioquímica Médica, Universidade Federal do Rio de Janeiro, Rio de Janeiro, Brazil; 2 Departamento de Genética, Universidade Federal do Rio de Janeiro, Rio de Janeiro, Brazil; 3 Centro de Ciências da Saúde, Universidade Católica de Petrópolis, Petrópolis, Brazil; 4 Hospital Municipal Jesus, Rio de Janeiro, Brazil; 5 Instituto de Puericultura e Pediatria Martagão Gesteira, Universidade Federal do Rio de Janeiro, Rio de Janeiro, Brazil; 6 Programa de Genética, Instituto Nacional de Câncer, Rio de Janeiro, Brazil; University Hospital Zurich, Switzerland

## Abstract

**Objective:**

Increasing evidence has accumulated showing the role of APOBEC3G (A3G) and 3F (A3F) in the control of HIV-1 replication and disease progression in humans. However, very few studies have been conducted in HIV-infected children. Here, we analyzed the levels of A3G and A3F expression and induced G-to-A hypermutation in a group of children with distinct profiles of disease progression.

**Methodology/Principal Findings:**

Perinatally HIV-infected children were classified as progressors or long-term non-progressors according to criteria based on HIV viral load and CD4 T-cell counts over time. A group of uninfected control children were also enrolled in the study. PBMC proviral DNA was assessed for G-to-A hypermutation, whereas A3G and A3F mRNA were isolated and quantified through TaqMan® real-time PCR. No correlation was observed between disease progression and A3G/A3F expression or hypermutation levels. Although all children analyzed showed higher expression levels of A3G compared to A3F (an average fold of 5 times), a surprisingly high A3F-related hypermutation rate was evidenced in the cohort, irrespective of the child's disease progression profile.

**Conclusion:**

Our results contribute to the current controversy as to whether HIV disease progression is related to A3G/A3F enzymatic activity. To our knowledge, this is the first study analyzing A3G/F expression in HIV-infected children, and it may pave the way to a better understanding of the host factors governing HIV disease in the pediatric setting.

## Introduction

Human immunodeficiency virus type 1 (HIV-1) infection in children often progresses rapidly to acquired immunodeficiency syndrome (AIDS). The majority of infected children under 15 years of age develop AIDS, and most die [1]. The risk of pediatric disease progression in mother-to-child transmission (MTCT) has been associated with different factors, such as mother with advanced HIV-1 disease, as well as immunological, virological and host-specific factors [2], [3].

A new class of host restriction factors has been described to play an important role in restricting intracellular viral replication. Enzymes of the apolipoprotein B mRNA-editing catalytic polypeptide (APOBEC) family convert cytidine to uridine in the transient (−)ssDNA replication intermediate, which is later reflected as G-to-A changes in the (+)strand [4], [5], [6], [7]. As these mutations usually occur at very high frequency in sequences, this phenomenon is often called hypermutation. Dinucleotide motifs within DNA are preferentially targeted by APOBEC3G (A3G) and APOBEC3F (A3F), resulting in proviral DNA GG to AG and GA to AA substitutions, respectively. A3G and A3F exhibit potent anti-HIV-1 activity [8], [9], [10], [11], [12], [13] and are expressed in lymphocytes, the major target cells for HIV-1 infection [9], [11]. HIV-1 Vif counteracts A3G/F by preventing their encapsidation into virions and by inducing their proteossomal degradation in the producer cell [14], [15]. The effects of A3G/F proteins likely modify both the efficiency of HIV-1 transmission and disease progression [16], but their role in these processes is not well understood.

A3G has been referred to as the major contributor to hypermutation *in vivo* and *in vitro* [12], [13], [17], [18], [19], [20], [21], [22], [23]. However, depending on the HIV genomic region analyzed, varying effects of A3G can be found. For example, hypermutation analyses in the HIV protease (PR) region of the *pol* gene have indicated a proportionally large number of G-to-A changes associated to A3F [24], [25].

Evidence suggests that individuals who express high levels of A3G mRNA tend to control the levels of HIV-1 viremia [26], [27]. It has also been suggested that levels of A3G expression decrease with disease progression [28]. A recent study showed that the frequency of hypermutated viral genomes in elite suppressors was not significantly different from that observed in patients on highly active antiretroviral therapy (HAART), and no correlation was found between A3G expression and the rate of hypermutation [20]. Differently from the previous report, the level of hypermutation was correlated with A3G expression in other studies [28], [29].

In the present study, we analyzed A3G and A3F mRNA expression levels, as well as G-to-A hypermutation contexts in HIV-1 proviral protease sequences from infected children with different profiles of disease progression.

## Methods

### Ethics Statement

All parents/guardians of the children read and signed an informed consent to participate to this study. This study was approved by the Institutional Review Board of the Federal University of Rio de Janeiro.

### Patients and Samples

Seventeen HIV-1-infected children with different profiles of disease progression regularly followed-up at the Pediatric Unit of Federal University of Rio de Janeiro since birth were enrolled in this study. All clinical and laboratory data, including CD4^+^ T-cell counts and HIV-1 viral load at every 3–4 months, were retrieved from a previous prospective study. All patients have been infected perinatally and therefore had known time of infection. Stored peripheral blood mononuclear cell (PBMC) samples were thawed and used for mRNA and proviral DNA extraction (see below). Six children were classified as long-term nonprogressors (LTNP), defined as over 8 years of age, with CD4≥25%, and under no antiretroviral therapy, a well-accepted definition for pediatric patients [30]; the remaining children (11 patients) were classified as typical progressors. Demographical, clinical, laboratory and follow-up data for all pediatric patients are summarized in Table 1. For A3G and A3F expression level determination, nine samples of HIV-negative children (NC) from the same previous study were used as a negative control group to assess A3G/F expression in the absence of HIV infection.

**Table 1:** Demographic and clinical characteristics of HIV-infected children under study.

Patient ID	Age*	Treatment experience*	CDC classification*	Follow-up Data			Sample Data		
				Period	Highest logHIV-1 VL	LowestCD4 T-cell%	Date	Log HIV-1 VL	CD4^+^T-cell%
N35/LTNP	10.2	No	A1	1994–2003	4.59	28	04/2003	3.73	27
N52/LTNP	10.6	Yes	A1	1992–2002	4.88	27	11/2002	3.88	33
N64/LTNP	11.3	No	A1	1993–2004	4.40	31	05/2003	4.08	33
N70/LTNP	13.7	No	A1	1996–2004	4.30	30	11/2003	4.34	32
N72/LTNP	7.7^#^	No	N1	2000–2007	2.58	29	12/2003	2.23	30
N82/LTNP	6.8^#^	No	N1	1998–2007	4.91	33	07/2004	4.85	34
N48/P	6.3	Yes	A3	2001–2004	4.28	17	02/2004	3.64	19
N58/P	5.8	No	A2	2000–2004	5.49	15	07/2003	5.49	25
N59/P	5.6	Yes	A2	2000–2005	5.04	5	09/2003	4.75	14
N61/P	12.2	Yes	A2	1998–2004	4.77	15	02/2003	2.86	23
N67/P	8.2	Yes	B2	1999–2005	5.28	10	04/2004	2.34	10
N75/P	6.8	Yes	B2	2000–2004	5.15	19	08/2003	1.96	22
N25/P	3.4	Yes	C3	2001–2003	6.45	1	06/2003	5.26	19
N27/P	3.0	Yes	C3	2001–2003	6.41	9	10/2002	5.30	15
N28/P	3.3	Yes	C3	2001–2003	6.97	6	09/2002	5.79	24
N33/P	3.0	Yes	C3	2001–2003	7.53	4	12/2003	4.83	35
N62/P	1.9	Yes	B3	2002–2003	5.49	6	12/2002	5.23	8

*At sample collection.

#Both patients had CD4%>25% at the age of 8 years old.

### HIV proviral DNA hypermutation

Genomic DNA from PBMC was isolated using the Illustra Blood GenomicPrep Mini Spin Kit (GE Healthcare). Nested PCR was then performed to amplify the HIV-1 proviral protease (PR) genomic region corresponding to HXB2 coordinates 2268–2564, as previously described [31]. HIV PR region was chosen because the first half of the *pol* gene is a highly frequent target of hypermutation-associated changes [21], [23], [32]. PCR-amplified products were cloned into pMOSBlue Blunt-ended PCR cloning kit (GE Healthcare) and a minimum of 10 clones from each patient were isolated by colony PCR and sequenced, therefore providing a sensitivity of 10% for detecting hypermutated sequences. Hypermutation was quantified using Hypermut 2.0 [33]. A sequence was considered hypermutated when the *p-*value was ≤0.05 in the Fisher's exact test that compared the number of G-to-A mutations by A3G/F *versus* a control context. In order to avoid overestimation of hypermutation by comparing different viral strains, we have used the plasma-derived genomic viral sequence generated by RT-PCR, representative of each patient's circulating virus, as the control context. We have also used the HIV-1 clone HXB2 as a reference for context in Hypermut for comparison of results. All patients were infected with HIV-1 subtype B viruses, as determined by phylogenetic analysis (not shown). All plasma-derived bulk sequences and proviral clonal sequences analyzed were submitted to the GenBank sequence database and were assigned the accession numbers JF950037 to JF950234.

### APOBEC3G and 3F mRNA expression analyses

Patient's PBMC also had their RNA extracted and purified for A3G and A3F mRNA expression analyses through TaqMan®-based real time PCR. Total RNA was extracted with Trizol®, precipitated with lithium chloride and quantified in a Nanodrop® apparatus. Two micrograms of RNA were subject to cDNA synthesis with Superscript RT and oligo-d(T) (Invitrogen, CA). For a subset of the samples, we have also primed the RT reactions with random hexameric primers, using the same amount of RNA. Primers and probes for A3G, A3F and the internal control GAPDH were synthesized through the Assay-on-Demand^TM^ (Applied Biosystems). Real time PCR reactions were conducted in an ABI 7500 apparatus (Applied Biosystems) in triplicate and with three different dilutions for each sample for determination of the reaction efficiency. Only reactions with efficiency superior to 95% were used in the calculations. Results were expressed as the relative number of A3G or A3F mRNA copies per 10^4^ copies of GAPDH mRNA.

### Statistical analyses

Demographic and laboratory data (CD4^+^ T-cell counts and HIV viral load) were compared among the two patient groups (typical progressors *versus* non-progressors) using Student's *t* tests, whereas for A3G and A3F expression level comparisons the Mann-Whitney *U* test was used. The definition of HIV-1 hypermutated sequences conducted in Hypermut was implemented within the software package using Fisher's exact tests. We have also compared A3G and A3F mRNA expression levels between patients for which hypermutation was detected and those with no hypermutation. Finally, we have stratified patients according to HIV viral load levels (below and above 10,000 copies/ml) and according to their treatment status (treatment-naïve or experienced), and compared the groups with respect to A3G/F expression. Calculations were performed in StatsDirect v.2.7.2 [34]. In all cases, *p*-values were considered significance at the ≤0.05 level.

## Results

Demographic and clinical characteristics of the HIV-infected children analyzed in this study are depicted in Table 1. Among the LTNP children, the lowest CD4^+^ T-cell percentage values during all time of follow-up ranged from 27 to 33%, whereas for progressors they ranged from 1 to 19%. The average age of the groups at the time of sample collection was 10.0 for LTNP and 5.4 for progressors (p = 0.005). As expected, CD4^+^ counts were higher among LNTP (p<0.001) when compared with the progressor group, but there was no difference in HIV viral load between the 2 groups (p = 0.50). Children of the negative control group (NC) averaged 4.7 years of age, ranging from 0.1 to 11.2 years (not shown).

APOBEC3G and 3F G-to-A hypermutation events in HIV proviral sequences were examined in the two disease progression groups of HIV-infected children. An alignment of all proviral clonal sequences referenced by their respective plasma viral bulk sequence can be seen in Figure S1. Hypermutated proviral sequences were detected more frequently among LTNP patients (50%) when compared with the P group (27%) (Table 2 and Figure S1), yet this difference was not statistically significant. Although less frequently, the percentage of clones with hypermuted sequences was higher in the P group patients. Results obtained using HXB2 instead of the patient's consensus plasma HIV sequence as reference for hypermutation determination were not significantly different (not shown). We could not find a clear correlation between hypermutation and the different patterns of disease progression. In both groups, the most frequent changes were GA to AA, surprisingly indicating a predominance of A3F-associated hypermutation (Table 2 and Figure S1). The hypermutated clonal viral sequence with the lowest *p*-value (higher significance) obtained in Hypermut from all patients had evidence of simultaneous A3G and A3F activity with the exception of sample N52-5, for which only A3G activity was detected (Figure S1).

**Table 2:** Frequency of G-to-A hypermutation in HIV-1-infected children with distinct profiles of disease progression.

PATIENTGROUP	NO. OFPATIENTS	NO. OFCLONES	HYPERMUTATION (%)		AVERAGE G-to-A CONTEXT (%)	
			Patients	Clones	GG (A3G)	GA(A3F)
LTNP	6	60	3	1 (10)	50	0
				1 (10)	37	83
				1 (10)	39	91
**Total**	**6**	**60**	**50%**	**10%**	**42%**	**58%**
P	11	121	3	2 (20)	35	5
				6 (60)	26	86
				1 (10)	32	13
**Total**	**11**	**121**	**27%**	**30%**	**31%**	**35%**

**Figure 1:**
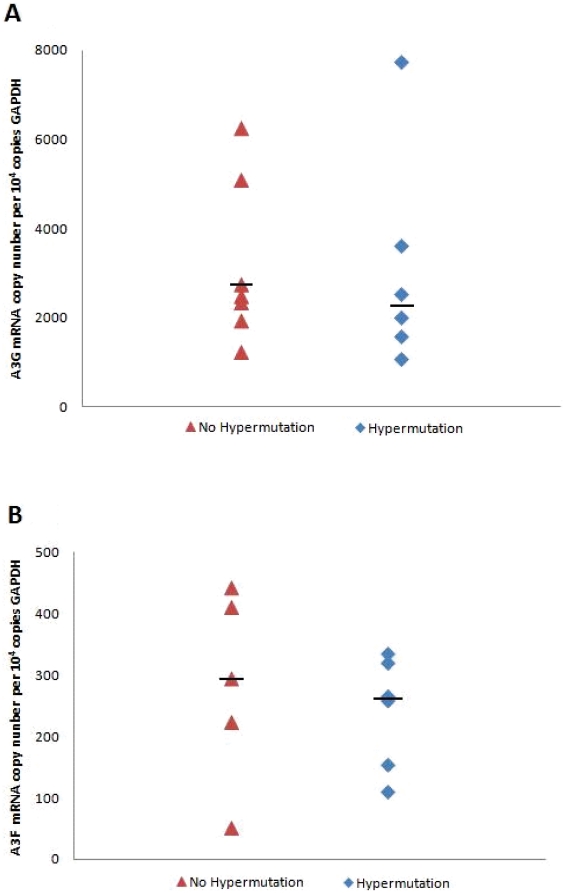
APOBEC3G (*A*) and APOBEC3F (*B*) mRNA expression levels relative to GAPDH mRNA copy numbers of patients with or without evidenced hypermutation. Horizontal bars depict the median value for each group.

We next investigated whether the G-to-A hypermutation changes were related to the levels of A3G and A3F mRNA expression. All patient samples with detected hypermutation (n = 6), as well as seven additional, randomly chosen samples of children with no signs of hypermutation, were selected for expression analysis. In all samples, A3G expression was higher than that of A3F (*p*<0.0001; Mann-Whitney *U* test), with fold variations ranging from 5 to 30 X (Figures 1A and B). Levels of both A3G and A3F expression did not differ significantly when comparing subjects with detected hypermutation with those where no hypermutation was detected (*p* = 0.84 and 0.66 for A3G and A3F, respectively; Mann-Whitney *U* test).

**Figure 2:**
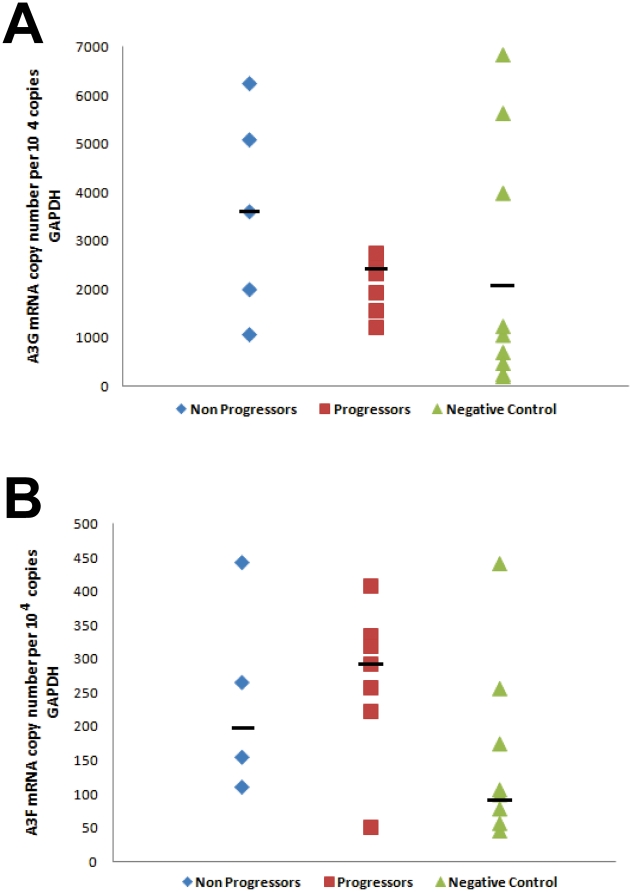
APOBEC3G (*A*) and APOBEC3G (*B*) mRNA expression levels relative to GAPDH mRNA copy numbers of patients with different profiles of HIV disease progression. Horizontal bars depict the median value for each group.

We wanted to further assess whether A3G and A3F expression levels were correlated with disease progression in HIV^+^ children. Nine samples of uninfected children were used as a negative control (NC) group. Results for A3G and A3F expression in each group are depicted in Figures 2A and B, respectively. There was no clear correlation between both A3G and A3F expression and disease progression in any group comparison done. Of note, when median A3G expression values were compared among groups, we found LTNP to be the highest A3G expressers (3600±2131/10^4^ cp GAPDH), followed by progressors (2626±1809) and NC (1049±2302). In our casuistic, the three highest A3G-expressing uninfected children had median expression values significantly higher than the median of the remaining NC (*p* = 0.02; Mann-Whitney *U* test). With respect to A3F expression, the progressors group was the higher expresser (270±113/10^4^ cp GAPDH), followed by LTNP (243±148) and NC (157±134). However, none of the median expression values was significantly different when the disease progression groups were compared. Of interest, we also failed to detect a significant correlation between A3G and A3F expression levels in both HIV^+^ and NC children groups (data not shown; R^2^ = 0.16 and 0.03, respectively).

**Figure 3:**
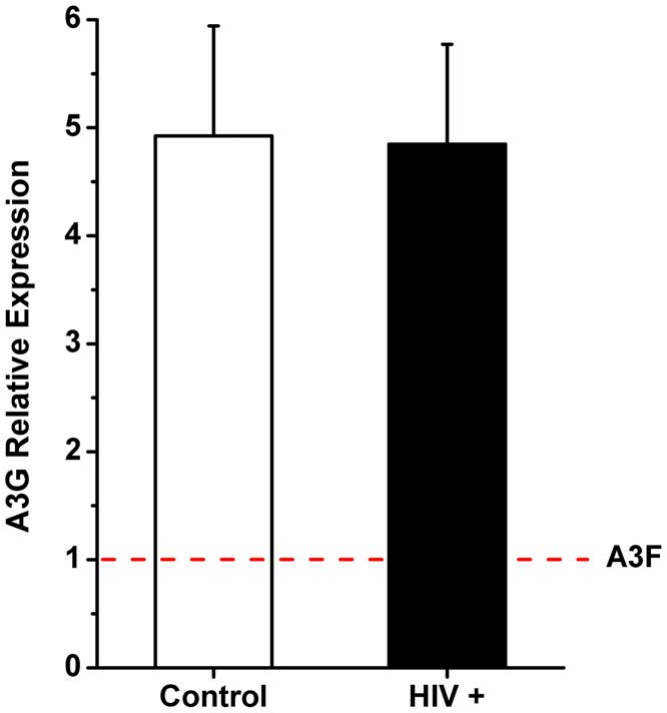
Average APOBEC3G to APOBEC3F mRNA expression levels quantified from eight HIV^+^ children (6 progressors and 2 nonprogressors) and five HIV^−^ controls. A3F expression levels were arbitrarily set at 1.

Recent reports have indicated that A3F mRNA expression levels might be largely underestimated when one uses oligo-d(T)-primed cDNA synthesis, due to the presence of repetitive elements in the long 3′ untranslated region of its mRNA [35]. To test for that possibility, we have subjected a subset of our patient's samples to random hexameric primer-based cDNA synthesis, which is reported to be more efficient under these circumstances [35]. Total RNA from plasma of 6/11 progressors, 2/6 nonprogressors and 5/9 HIV-negative controls were re-synthesized with random primers and subject to the same mRNA A3G and A3F quantification protocol. Indeed, the fold difference between A3G and A3F changed significantly, with an average fold difference of 5x more A3G compared to A3F (range 2–9X) (Figure 3), as opposed to the 15x fold difference seen with oligo-d(T)-primed reactions. These results suggest that A3F mRNA levels were underestimated in the previous experiments. However, the new procedure did not change the relative profiles of A3G and A3F expression among different groups, with HIV^+^ subjects displaying higher expression levels than negative controls (data not shown). The differences observed between groups were not statistically significant, as in the previous experiments.

**Figure 4:**
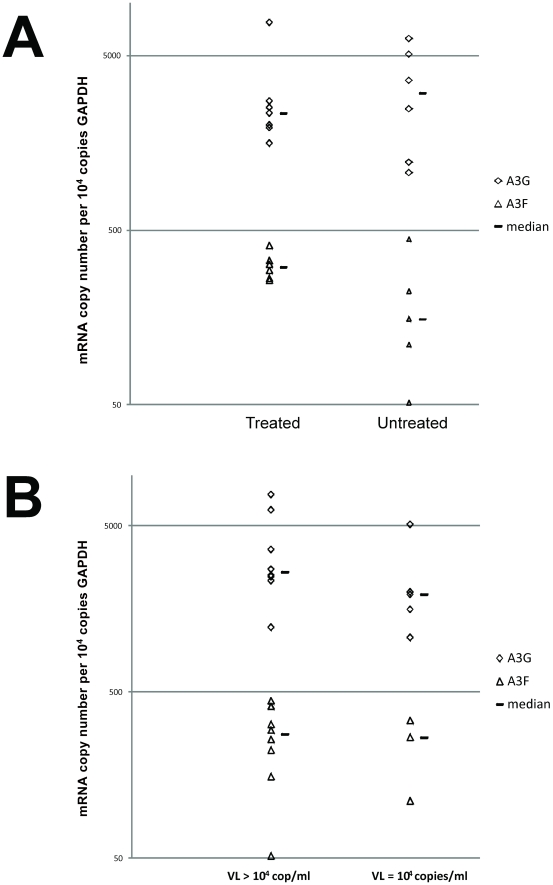
APOBEC3G and APOBEC3F mRNA expression levels relative to GAPDH mRNA copy numbers comparing patients stratified according to antiretroviral treatment exposure (*A*) and HIV viral load (*B*). Horizontal bars depict the median value for each group.

We finally assessed the effect of antiretroviral treatment and of the HIV-1 viral load levels on A3G/F mRNA expression in our casuistic. When children were categorized in treated *versus* untreated, irrespective of their disease progression profile, there were no significant differences in the levels of either A3G or A3F mRNA expression levels (Figure 4A; p = 0.80 and 0.16, respectively). Children with low HIV VL (below 10,000 copies/ml) also did not show different levels of A3G/F expression when compared to those with VL above that cut-off (Figure 4B; p = 0.25 and 0.71, respectively).

## Discussion

Previous studies have addressed G-to-A hypermutation and APOBEC3G sequence variations on disease progression in infected children [36], [37]. Our study, however, describes for the first time the analysis of APOBEC3G and 3F mRNA expression levels in an infected pediatric setting. Here, we investigated whether hypermutation and APOBEC expression were associated with different patterns of disease progression in this small cohort of pediatric patients.

We were unable to correlate HIV hypermutation levels to disease progression in children. Although our study has included a small number of individual proviral sequences from each patient, the lack of correlation between delayed disease progression and hypermutation found here is in agreement with recent findings in adults. Gandhi *et al*. found no correlation between hypermutation in elite suppressors when compared to that in HIV^+^ patients undergoing HAART, after analyzing a large number of HIV-1 sequences [20]. This observation suggests that hypermutation levels are not directly related to the intrinsic control of viremia seen in LTNP. Indeed, we found one LNTP child with low HIV viral load (3.73 log^10^/mL of plasma) in which no hypermutation was detected. Conversely, two samples from progressors, with hypermutated sequences, had HIV VL over 5 log_10_/mL. Our results further suggest a lack of correlation between HIV VL and hypermutation (data not shown). While some other reports have also failed to correlate HIV VL with either A3G-mediated hypermutation [29], [38] or to A3G expression levels [20], [26], [27], [39] in adults, different studies showed correlation between A3G mRNA expression or G-to-A hypermutation and disease progression [28], [32], and this issue is warranted further investigation.

Of interest, we have found a high contribution of A3F-mediated hypermutation in the HIV-1 PR *pol* genomic region of viruses from our children, a finding previously reported in infected adults. A recent study by Armitage *et al*. has shown that, whereas A3G hypermutates HIV proviruses roughly evenly across the genome, A3F promotes higher numbers of changes at definite regions, the largest being the *pol* PR region [23]. Indeed, A3F-mediated hypermutation in HIV PR has been previously described [9], [24]. These observations might explain, at least in part, the relatively high observation of A3F-mediated hypermutation events in our patients.

A re-analysis of A3G and A3F expression levels with random primed-cDNA synthesis in a subset of HIV^+^ and negative control children for which cellular RNA was still available showed that the A3F levels were underestimated, with fold differences to A3G levels three times lower than those estimated with oligo-d(T)-primed reactions. This is in agreement with data of Refsland et al. [35], which suggest that the long A3F mRNA 3′UTR presents repetitive elements that can impair oligo-d(T)-primed cDNA synthesis. Moreover, our confirmatory results further corroborate the importance of A3F-mediated hypermutation in the pediatric cohort studied.

Cho *et al*. have previously reported a positive correlation between the expression of A3G and A3F in adults [39]. In our pediatric casuistic, we failed to detect such correlation, both in HIV^+^ and in uninfected children. It is plausible that the differences observed between their study and ours are related to the age groups analyzed. Children's immune systems are not completely developed, and HIV infection during development may also impair specific immune components. A3G and A3F expression levels might not correlate well in a partially-developed immune system. In this view, it has been shown that distinct CD4^+^ T-helper lymphocyte subtypes show differences in the levels of A3G expression [40]. Th1 effector cells express higher levels of A3G and A3F mRNA, and of A3G proteins, than Th2 cells. In addition, HIV-1 virions produced in Th1 cells carry increased amounts of incorporated A3G compared to those produced in Th2 cells [40]. As a result of lower ratios of CD4^+^ Th1 cells observed in children compared to adults [41], children might have a proportionally higher A3F-mediated hypermutation activity. Finally, differential expression of the A3 proteins has been shown in different human tissues [35]. In particular, tissues such as the thymus and the spleen, which carry a large amount of T cells, and have significantly different activities in children and adults, can explain discrepancies in the levels of A3G and A3F seen in both groups.

We cannot rule out the possibility that differences in A3G and A3F protein activity are due to amino acid polymorphisms, to protein stability or to differential interaction with Vif taking place in the individuals analyzed here. Indeed, a number of Vif mutants have been recently shown to lack A3G-counteracting activity, and even interfere with wild type Vif molecules [42]. Conversely, amino acid changes in several A3 coding regions, including A3G and A3F, also alter susceptibility to HIV-1 Vif [43], [44]. Additional studies in the A3G and A3F coding sequences will improve our understanding of the role of each protein in hypermutation. At the protein level, it is recognized that A3G exists in two forms within cells, aggregated into low (LMM) or high molecular mass (HMM) complexes [45]. While the first form is active in virus deamination, the second is inactive. It has been shown that selected cytokines, like IL-2, IL-7 and IL-15, induce a shift of A3G into HMM complexes, rendering CD4^+^ T-cells more permissive to HIV infection. Other cytokines, such as TNFα, upregulate A3G in cells but leave them in LMM complexes, active against HIV [45]. Again here, differences in the immune system of children compared to that of adults might account for discrepancies in the amount of enzymatically-active forms of A3 proteins.

Another important issue to be considered here is the fact that many different A3 proteins display anti-HIV activity, as reviewed by Albin and Harris [46]. Although A3G and A3F have been recognized as major players in HIV restriction, other members of the APOBEC family such as A3DE and A3H have been shown to harbor anti-HIV activity, as well as sensitivity to lentiviral Vif proteins [43], [46], [47]. In this scenario, we cannot rule out the possibility that other A3 proteins with similar target contexts to A3F, particularly A3H [48], [49], are contributing to the higher non-A3G-mediated hypermutation seen herein.

Previous studies have reported higher levels of A3G expression in subjects infected by HIV or HCV compared to uninfected individuals [27], [50]. Our results are in agreement with those studies, and extend this observation to A3F. In our control group, however, the three highest A3G-expressing uninfected children had median expression values significantly higher than the median of the remaining NC, suggesting that A3G expression may vary substantially between individuals depending on various unrelated infections and on other immunological factors. It has been recently shown that infection by influenza A virus upregulates A3G, but not A3F expression in infected cells [51]. Therefore, the possibility that varying, unrecognized clinical conditions might alter A3G expression levels in individuals cannot be ruled out, and may prevent the establishment of clear associations between HIV infection and A3G expression.

We failed to observe any association between HIV VL or treatment experience and A3G/F mRNA expression in our patients. This is agreement with the report by Ulenga *et al*., which were not able to correlate hypermutation with VL in infected adults [29]. A previous report failed to find differences in A3G-mediated hypermutation between patients with undetectable VL by natural means (among elite supressors) or with the use of antiretroviral therapy [20], indicating that treatment *per se* does not influence A3G expression levels. Further studies are warranted to define specific biological or chemotherapeutical circumstances under which APOBEC expression is modulated.

Overall, we were unable to find any correlations between A3G or A3F expression levels, A3G- or A3F-induced hypermutation and disease progression profiles in HIV-infected children. This is however, to the best of our knowledge, the first study of A3G and A3F mRNA expression conducted in pediatric HIV/AIDS patients. The study of different disease progression profiles in the pediatric setting is also worth mentioning. Interestingly, we found evidence of a more pronounced A3F activity, not previously reported in adults, that merits further investigation. We believe this study helps contributing to a better understanding of the role of restriction factors and host genetics in an infectious disease pediatric setting.

## Supporting Information

Figure S1 HIV-1 protease nucleotide alignments of bulk and clonal proviral sequences of each patient analyzed in the study. The bulk sequence of each patient is used as a reference at the top of each alignment. Dots represent nucleotide identities.
(TIF)
